# Cascaded discrimination of normal, abnormal, and confounder classes in histopathology: Gleason grading of prostate cancer

**DOI:** 10.1186/1471-2105-13-282

**Published:** 2012-10-30

**Authors:** Scott Doyle, Michael D Feldman, Natalie Shih, John Tomaszewski, Anant Madabhushi

**Affiliations:** 1 Ibris, Inc., Monmouth Junction, New Jersey, USA; 2 Department of Surgical Pathology, University of Pennsylvania, Pennsylvania, USA; 3 School of Medicine and Biological Sciences, Buffalo University, Buffalo, USA; 4 Department of Biomedical Engineering, Case Western Reserve University, Ohio, USA

## Abstract

**Background:**

Automated classification of histopathology involves identification of multiple classes, including benign, cancerous, and confounder categories. The confounder tissue classes can often mimic and share attributes with both the diseased and normal tissue classes, and can be particularly difficult to identify, both manually and by automated classifiers. In the case of prostate cancer, they may be several confounding tissue types present in a biopsy sample, posing as major sources of diagnostic error for pathologists. Two common multi-class approaches are one-shot classification (OSC), where all classes are identified simultaneously, and one-versus-all (OVA), where a “target” class is distinguished from all “non-target” classes. OSC is typically unable to handle discrimination of classes of varying similarity (e.g. with images of prostate atrophy and high grade cancer), while OVA forces several heterogeneous classes into a single “non-target” class. In this work, we present a cascaded (CAS) approach to classifying prostate biopsy tissue samples, where images from different classes are grouped to maximize intra-group homogeneity while maximizing inter-group heterogeneity.

**Results:**

We apply the CAS approach to categorize 2000 tissue samples taken from 214 patient studies into seven classes: epithelium, stroma, atrophy, prostatic intraepithelial neoplasia (PIN), and prostate cancer Gleason grades 3, 4, and 5. A series of increasingly granular binary classifiers are used to split the different tissue classes until the images have been categorized into a single unique class. Our automatically-extracted image feature set includes architectural features based on location of the nuclei within the tissue sample as well as texture features extracted on a per-pixel level. The CAS strategy yields a positive predictive value (PPV) of 0.86 in classifying the 2000 tissue images into one of 7 classes, compared with the OVA (0.77 PPV) and OSC approaches (0.76 PPV).

**Conclusions:**

Use of the CAS strategy increases the PPV for a multi-category classification system over two common alternative strategies. In classification problems such as histopathology, where multiple class groups exist with varying degrees of heterogeneity, the CAS system can intelligently assign class labels to objects by performing multiple binary classifications according to domain knowledge.

## Background

Digital pathology (DP) has allowed for the development of computerized image-based classification algorithms to be applied to digitized tissue samples. Recent research has focused on developing computer-aided diagnostic (CAD) tools that can classify tissues into one of two classes, such as identifying “cancer” vs. “non-cancer” tissues [1-5]. However, in the case of CaP, the “non-cancer” class includes various heterogeneous tissue types such as epithelium and stroma tissue as well as confounding classes such as atrophy, PIN, and perineural invasion. Ideally one would wish to employ a multi-class approach to distinguish between several different tissue types at once.

**Figure 1:**
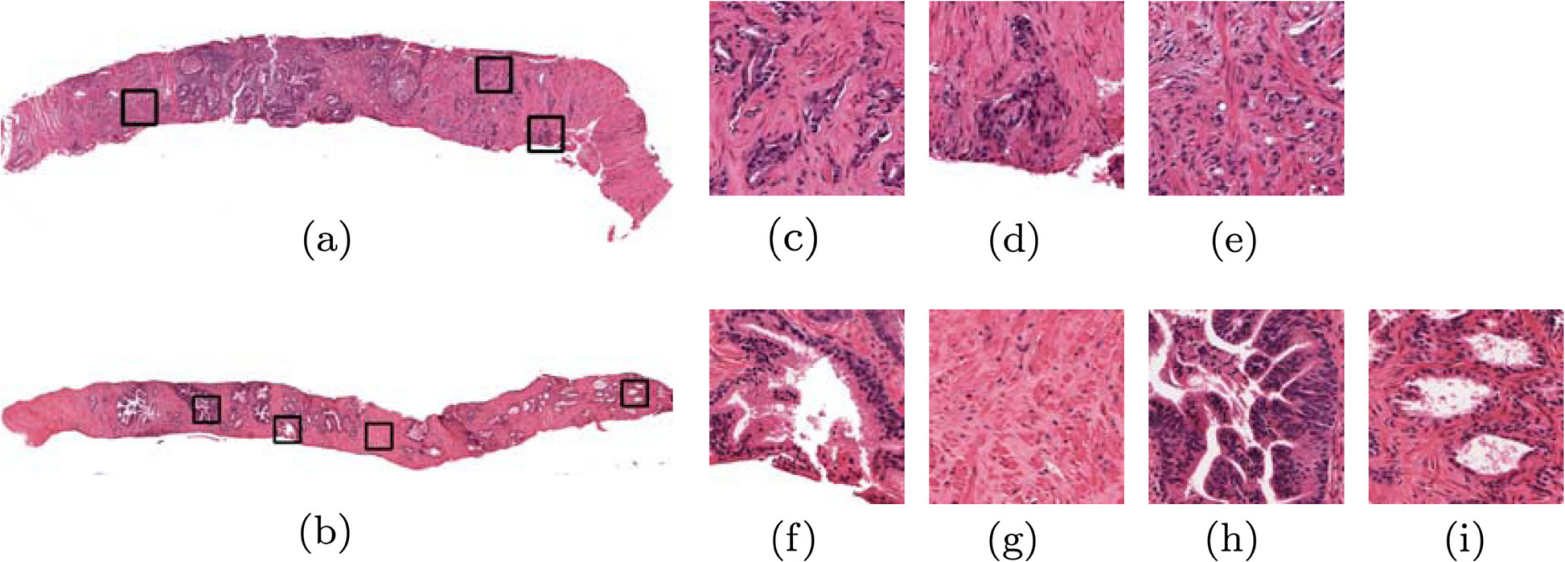
**Illustration of different prostate biopsy tissue types.** Shown are regions of interest (ROIs) taken from the whole-slide images shown in (**a**) and (**b**). The following tissue types are illustrated: (**c**) prostate cancer (CaP) Gleason grade 3, (**d**) CaP Gleason grade 4, and (**e**) CaP Gleason grade 5, normal tissue categories (**f**) benign epithelium and (**g**) benign stroma, and CaP confounders including (**h**) prostatic intraepithelial neoplasia (PIN) and (**i**) tissue atrophy. Note that atrophy and PIN can sometimes be mistaken for CaP and hence pose a diagnostic problem.

Over 240,000 new cases of prostate cancer (CaP) are expected to be diagnosed in the US in 2011 [6]. Blinded needle sextant biopsy is the current gold standard for CaP diagnosis, each biopsy procedure typically yielding between 12-15 tissue cores, each of which is examined under a microscope. If CaP is identified, a pathologist will then use the Gleason grading scale to identify aggressiveness based primarily on tissue architecture [7]. Examples of prostate tissue obtained via biopsy are shown in Figure 1; a single tissue core might comprise multiple tissue classes (e.g. normal, different Gleason grades of CaP, and confounders). Manual analysis of cancer is limited due to several factors: (1) The subjective, qualitative nature of Gleason grading leads to a high degree of inter-observer variability [8]. (2) Confounders, or non-cancerous tissue patterns that have attributes that are intermediate to normal and diseased processes can complicate diagnostic identification of tumor areas [9,10]. Apart from being able to discriminate confounders from disease, correct identification of confounders is important, as they may harbor useful diagnostic information [11]. (3) Manual analysis requires careful examination of each tissue sample; this becomes prohibitive in the case of saturation biopsies where between 32-48 tissue samples might be acquired during a single prostate biopsy procedure. (4) Physical tissue samples are not amenable to consultation by outside experts; transport of glass slides for second-opinion reading is expensive and time-consuming. 

Automated, computerized image analysis of histopathology has the potential to greatly reduce the inter-observer variability in diagnosis of biopsy samples [12-15], and algorithms have been developed for detecting neuroblastoma [15], quantification of lymphocytic infiltration on breast biopsy tissue [16], and grading astrocytomas on brain tissue [17], to name a few. In the context of CaP, researchers have used a variety of features to analyze tissue ranging from low-level image features (color, texture, wavelets) [18], second-order co-occurrence features [19], and morphometric attributes [1]. Farjam, et al. [20] employed gland morphology to identify the malignancy of biopsy tissues, while Diamond, et al. [21] used morphological and texture features to identify 100-by-100 pixel tissue regions as either stroma, epithelium, or cancerous tissue (a three-class problem). Tabesh, et al. [1] developed a CAD system that employs texture, color, and morphometry on tissue microarrays to distinguish between cancer and non-cancer regions, as well as between high and low Gleason grade prostate cancers (both cases used binary classification).

**Figure 2:**
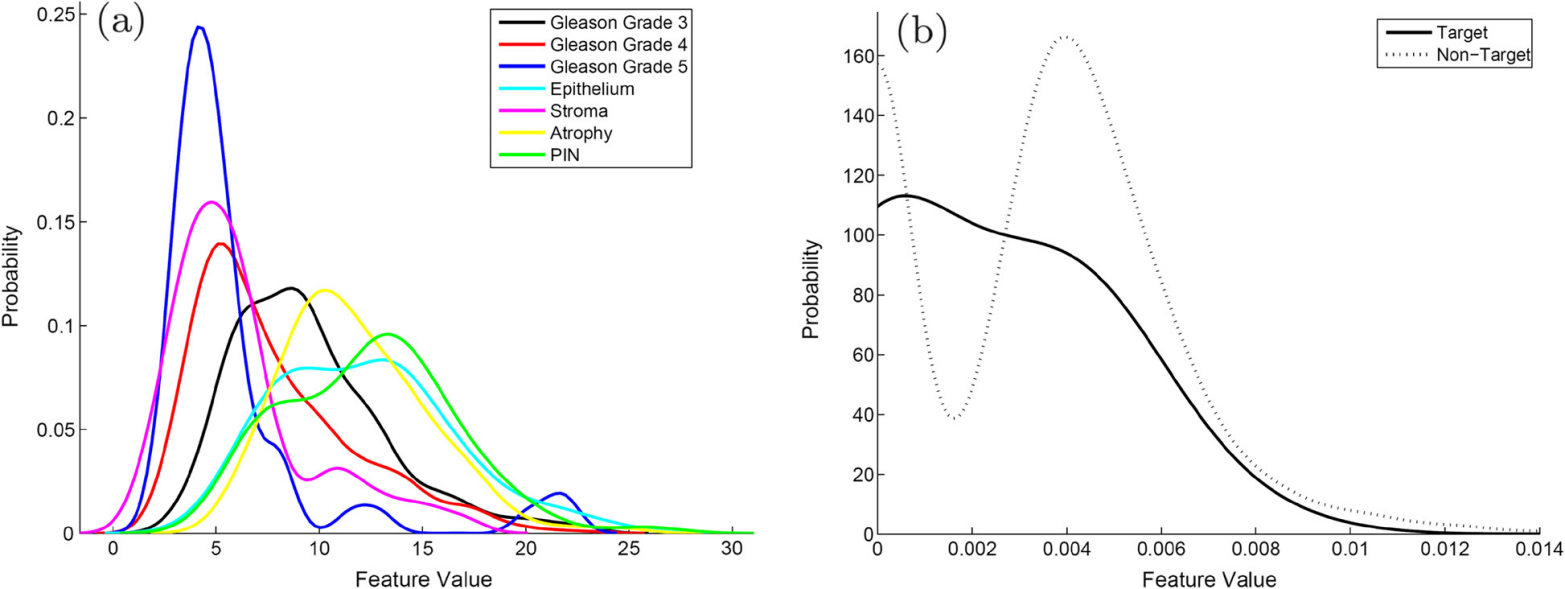
**Probability density functions for OSC and OVA.** Illustration of probability density functions, where the likelihood of observing a particular class (dependent axis) is plotted against a feature value (independent axis). Shown are two different multi-class strategies: (**a**) OSC, where all classes are plotted simultaneously, and (**b**) OVA, where a “Target” class is separated from a heterogeneous “Non-target” class. A heterogeneous set of tissues in the non-target class can lead to multi-modal density functions, illustrated by the dotted line.

There are two common approaches to the multi-category classification problem, as illustrated in Figure 2. The first is to perform one-shot classification (OSC) of several classes at once. These typically involve classifiers such as decision trees [22] that are inherently able to deal with multiple classes simultaneously. This approach is limited when dealing with multiple similar classes, since all classes must be distinguished simultaneously using the same classifier and the same set of features. Assigning multiple decision boundaries can lead to classification errors, particularly when some classes are similar (e.g. different types of cancerous tissue) and others are dissimilar (cancerous and benign tissue). An illustration of this type of classifier is shown in Figure 2(a), where each curve represents a probability density function, in turn reflecting the likelihood of observing a particular class for a specific image-derived attribute. Clearly, assigning a set of decision boundaries to separate out these classes would lead to suboptimal results. An alternative is the one-versus-all (OVA) approach, where each class is individually distinguished from all non-target classes. Figure 2(b) showcases this approach, where the “Target” class probability is plotted against the “Non-target” class. Since the non-target encompasses a number of visually diverse tissue classes, the non-target class distribution is multi-modal, and assigning a single classification boundary in this case would be sub-optimal. 

**Figure 3:**
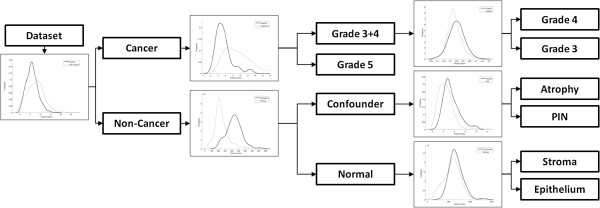
**Illustration of the cascaded (CAS) approach.** Each probability density function represents a single binary classification task. Beginning at the left, all images in the dataset are classified into either cancer or non-cancer categories. A new binary classification is then performed: cancer images are classified as Gleason grades 3/4 or grade 5, and non-cancer images are classified as confounder or normal images. The final set of binary classifiers separates the Gleason grade 3+4 group into G3 and G4 separately, confounder images are identified as AT or PIN, and normal images are identified as BS or BE.

A more strategic approach is to employ a cascaded scheme (CAS), as illustrated in Figure 3. In this strategy, successive binary classifications are performed where at each level in the classifier cascade, similar-appearing tissue classes are clustered together according to class similarity and domain knowledge. Previous work has shown that some classification tasks are more easily handled by dividing the original problem into separate sub-problems [23], which can then be tackled individually. Each bifurcation in Figure 3 represents a binary classifier that distinguishes dissimilar “class groups,” ensuring that the classes within a group are relatively similar. Subsequently, two new binary classifications are used to separate each of the class groups further, again grouping similar sub-classes together. At each level in the cascade the aggregated classes are broken down with increasing granularity into constituent subclasses, until at the lowest cascade level the individual constituent classes are separated from each other. The CAS approach is particularly well-suited to supervised classification problems in DP due to the existence of multiple nested categories of tissue types, and confers two distinct advantages over both OSC and OVA classification: (1) By utilizing multiple independent binary classifiers, we avoid the problem of having to identify multiple classes at once using the same classifier, and are thus able to tailor the classifiers to each pairwise classification problem by selecting features and parameters that are optimized for each particular binary classification problem. (2) By determining the class groupings based off domain knowledge, we are able to minimize the class heterogeneity for each classification task at each level in the cascade, thus avoiding the problem of trying to discriminate between classes with significant overlapping class distributions such as with the OVA approach. 

In this work, we apply the cascaded classifier in the context of classifying regions of interest (ROIs) of prostate tissue into one of seven classes: Gleason grades 3, 4, and 5 (abbreviated G3, G4, and G5, respectively), benign epithelium (BE), benign stroma (BS), tissue atrophy (AT), and PIN. From each ROI, a set of novel image features are extracted which quantify the architecture (location and arrangement of cell nuclei) and texture of the region. These feature vectors are used in a cascaded classification approach whereby similar classes are grouped according to domain knowledge, and binary classification is performed at increasing levels of granularity. We test our algorithm by comparing the cascaded approach with two traditional multi-class approaches: the OSC approach, where classification algorithms attempt to distinguish all classes simultaneously, and the OVA approach, where individual classes are classified independently from all other classes. We show that by incorporating domain knowledge and utilizing the cascaded classifier, we can more accurately identify nested subclasses.

This work is an extension of our previous work in identifying regions of cancer vs. non-cancer in prostate biopsy images on a pixel-by-pixel basis using a hierarchical classifier [5]. Our previous approach was developed to identify suspicious regions on very large images, using pyramidal decomposition until individual pixels could be classified as cancer or non-cancer. The major differences in the current work are the following: (1) we are classifying tissue regions as opposed to individual pixels, so our analysis and feature extraction are necessarily different, and (2) dealing with multiple categories of tissue types instead of the “cancer” vs. “non-cancer” question. Additionally, an important objective of this work is to illustrate the performance increase obtained by the CAS approach compared with OVA and OSC.

## Methods

### Cascaded multi-category classification

#### Notation and definitions used

An example of an annotated digital biopsy sample is shown in Figure 1, with zoomed in exemplars of each tissue class. Mathematically, we denote an ROI as $\left(R=(R,g)\right)$, where *R* is a 2D set of pixels *r*∈*R*and *g*(*r*) is an intensity function that assigns a triplet of intensity values to each pixel (corresponding to the red, green, and blue color channels of the image). The class of $\left(R\right)$ is denoted as *ω*_*i*_ for *i*∈{1,⋯ ,*k*} classes, and we use the notation $\left(R↪\omega_{i}\right)$ to indicate that $\left(R\right)$ belongs to class *ω*_*i*_. In this work, *k*=7.

#### Class groupings in cascaded classifier

To classify $\left(R\right)$, we employ the cascaded approach illustrated in Figure 3. The cascaded setup consists of a series of binary classifications, which divides the multi-category classification into multiple two-category problems. Each bifurcation in Figure 3 represents a separate, independent task with an independently-trained classifier, amounting to six binary divisions. The motivation for the chosen class groups is based on domain knowledge. The first bifurcation handles all the samples in the database, classifying them as “cancer” or “non-cancer” images. Within the cancer group, we further classify samples into either G5 or a class group containing G3 plus G4; this is done because within the cancer group, G3 and G4 are more similar to one another than either is to G5. (Note that in this paper, when we refer to “Gleason grades 3+4”, we are referring to the group of images that are members of either primary Gleason grade 3 or primary grade 4 CaP as opposed to images representing a Gleason pattern of 3+4, i.e. Gleason sum 7. All ROIs are considered to be homogeneous regions of a single tissue pattern.) Similarly, non-cancer samples are identified as either “confounder” classes, which contain abnormal but non-cancerous tissue, or “normal” class groups. Finally, each of the remaining class groups is further classified to obtain the final classification for all samples: the Gleason grade 3+4 group is separated into G3 and G4, the confounder images are classified as AT or PIN, and normal tissues are classified as BE or BS.

#### Cascaded decision tree classifier

For each binary classification task in the cascade, we use a decision tree classifier [22]. Decision trees use a training set of labeled samples to learn a series of rules or “branches” based on feature values. These rules attempt to optimally distinguish between each of the class labels, which are represented by “leaves” at the end of the tree. Classification can then be performed on a testing set, using the features of each testing sample to traverse the tree and arrive at the leaf representing the correct class of the sample. While any classification algorithm may be used in the framework of the cascaded classification, we chose to decision trees for a number of reasons: (1) Decision trees can inherently deal with several classes by creating multiple different class leaves, allowing us to implement the OSC classification strategy directly for comparison. (2) The structure of the tree can be examined to determine which features appear closest to the top of the tree, which are typically the most discriminating features for that classification task. Additionally, these features are selected independently for each of the classification tasks, allowing us to use an optimal set of features for each level of the cascade.

### Detection and segmentation of nuclei

#### Color deconvolution for nuclei region detection

**Figure 4:**
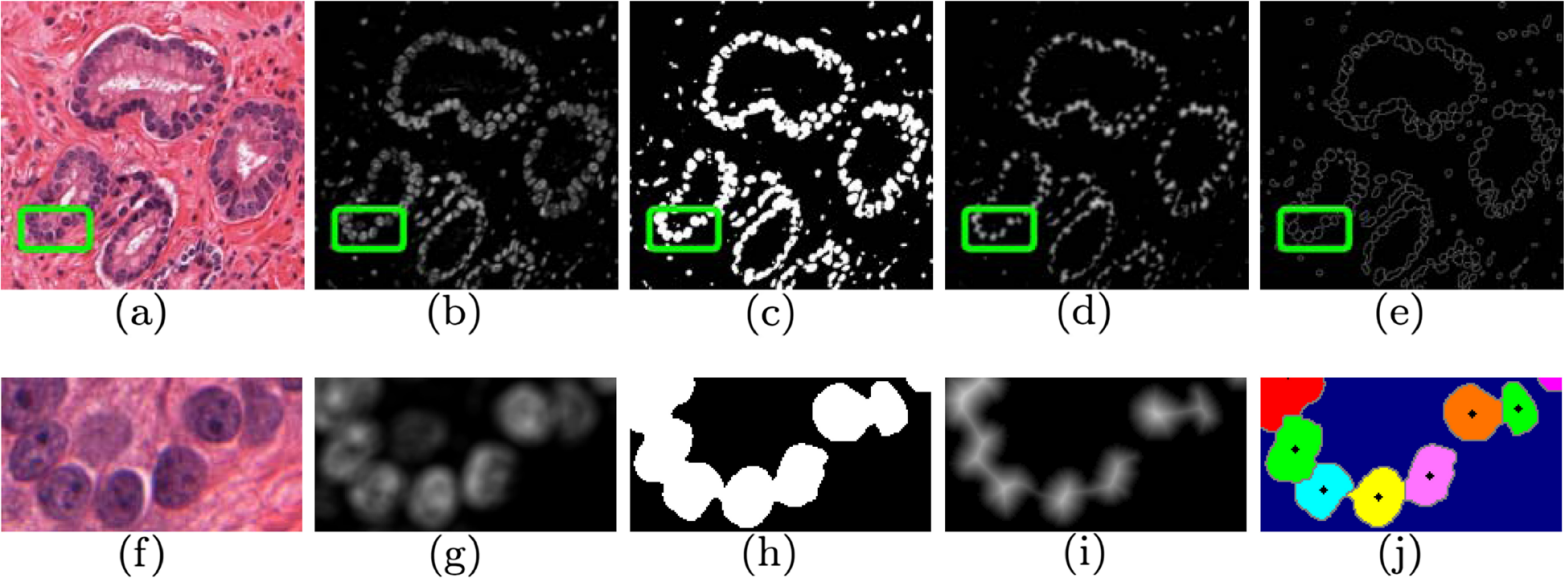
**Overview of automatic nuclei detection.** Shown are: (**a**), (**f**) the original tissue image, (**b**), (**g**) the result of color deconvolution to isolate the nuclear stain, (**c**), (**h**) the result of thresholding to get nuclear regions, (**d**), (**i**), the result of the Euclidean distance transform on the thresholded image, and (**e**), (**j**) the result of watershed segmentation of the nuclear boundaries. In (**j**) the different regions have been marked in color, and the nuclear centroids have been labeled.

To isolate nuclear regions we use a color deconvolution process detailed in [24]. The optical density (OD) of a material is given by $\left(a=-\log\frac{I}{I_{0}}\right)$, where *I* is the intensity of light transmitted through the material (i.e. the light detected by the microscope or scanning hardware), and *I*_0_is the intensity of light incident on the material [25]. The value of *a* can be found empirically by measuring the incident and transmitted light for each channel (red, green, and blue) and each stain of an image. We obtain a normalized three-by-three matrix **M**where the rows indicate the materials in the sample (hematoxylin, eosin, and background) and the columns denote the red, green, and blue channels of the image. We denote by **C**the three-element vector representing the amount of each stain at a pixel *r*, then *g*(*r*)=**CM** represents the three-element intensity vector at *r*. We can then solve **C**=*g*(*r*)**M**^−1^to obtain the amount of each stain present at pixel *r*[24]. Shown in Figures 4(a), (f) are tissue samples, followed by the result of color deconvolution in Figure 4(b), (g) where the intensity of the pixels is proportional to the amount of hematoxylin stain present. Shown is the channel corresponding to the hematoxylin stain (the nuclear material). 

#### Finding nuclear centroids via watershed segmentation

The deconvolved image shows the relative amount of stain at each pixel. To obtain the nuclear centroids, denoted *v* ∈ *V*, we employ a watershed algorithm [26] to segment the nuclear region, and find the centroids of the resulting connected components. The watershed algorithm is a method of segmenting an object by assuming that high values of $\left(D\right)$ are “valleys” that can be filled with water, and local maxima are point sources [27]. The points where two pools merge are considered the segmentation of the region, and the set of nuclear centroids *V * is then derived from the geometric center of the segmented nuclei. We perform the following steps: 

1. Binarize the image using Otsu’s thresholding method [28] to yield the set of pixels within the nuclear region, denoted *N*.

2. The set of pixels on the boundary of *N* (immediately adjacent) are denoted *C*, *N* ∪ *C* = *∅*.

3. The Euclidean distance transform is applied to the binarized image to generate a distance map $\left(D=(R,d)\right)$, where *d*(*r*) is the distance from pixel *r* to the closest point on *C*.

4. Local maxima in $\left(D\right)$ are identified as the start points for the watershed algorithm, which iterates until all pixels in *N* are segmented.

Shown in Figure 4 are examples of the watershed algorithm’s steps, including the binarized image (Figures 4(c) and 4(h)), the distance map $\left(D\right)$ (Figures 4(d) and 4 (i)), and the resulting watershed contours (Figures 4(e) and 4(j)). Different colors in Figures 4(e) and (j) indicate different pools or segmentations, and black dots indicate the centroids of the detected regions.

### Quantitative image feature extraction

From each image, we extract a set of nuclear architecture features as well as image texture features, described in detail in the following sections. A summary list of the features used in this study can be found in Table 1.

**Table 1:** List of features

**Feature type**	**Feature subtype**	**Features**	**Total**
Architecture	Voronoi Diagram	Area, chord length, perimeter	12
	Delaunay Triangulation	Area, perimeter	8
	Minimum Spanning Tree	Branch Length	4
	Nuclear Density	Nearest Neighbors, distance to neighbors	24
Texture	First-Order	Statistics, Sobel and Kirsch filters, Gradients	135
	Co-occurrence	Autocorrelation, Contrast, Correlation, Cluster	189
		Prominence, Cluster Shade, Dissimilarity,	
		Energy, Entropy, Homogeneity, Maximum	
		probability, Variance, Sum average, Sum	
		variance, Sum entropy, Difference variance,	
		Difference entropy, two information measures	
		of correlation, Inverse difference, Normalized	
		inverse difference, inverse difference moment	
	Steerable Filter	Frequency and Orientation Parameters	216

List of the features used in this study, broken into architectural and texture features.

#### Nuclear architecture feature extraction

**Figure 5:**
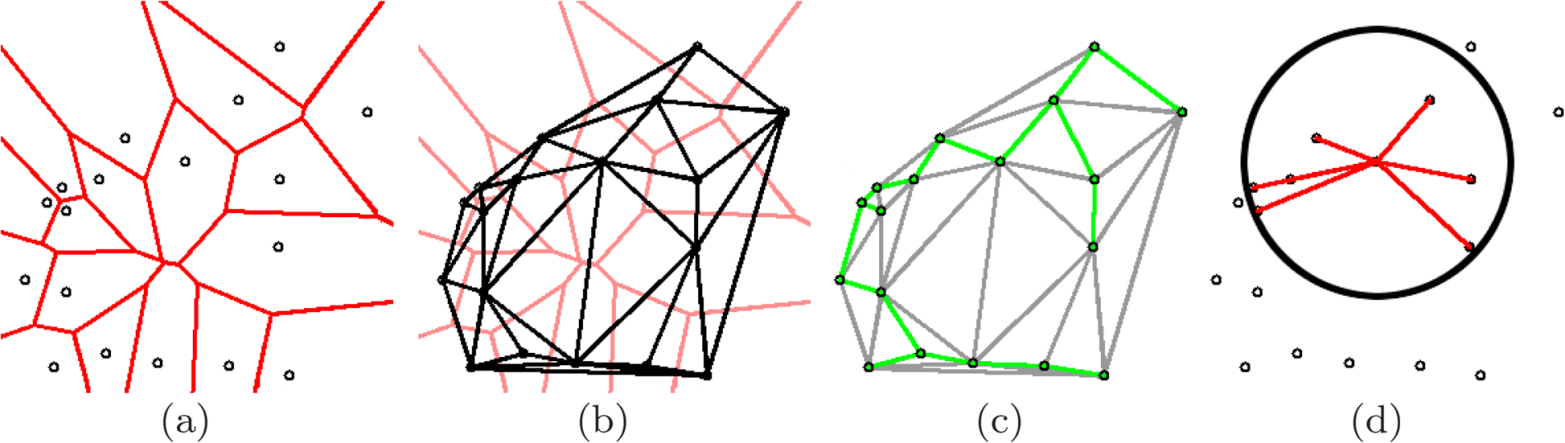
**Architectural pattern graphs.** Examples of the graphs used to quantify architectural patterns in digital tissue. From a series of nuclear centroids (represented by black circles), we create (**a**) the Voronoi Diagram (red), (**b**) the Delaunay Triangulation (black), and (**c**) the Minimum Spanning Tree (green), as well as (**d**) density statistics of a neighborhood represented by the thick black circle. Red lines in (**d**) represent the distance from the point of interest (upon which the neighborhood is centered) to all other points in the neighborhood.

**Figure 6:**
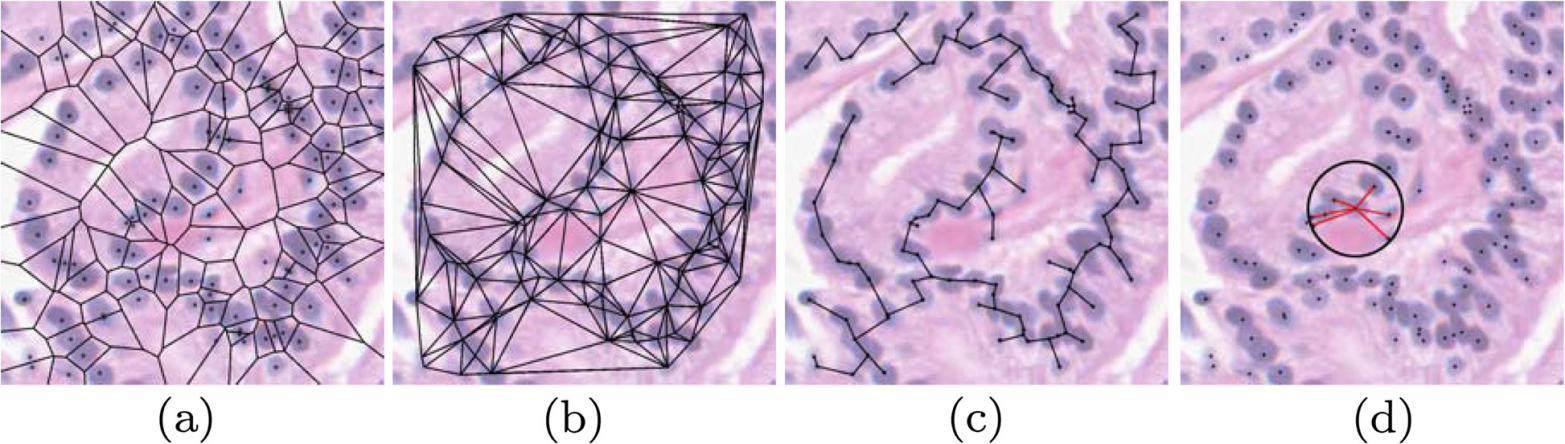
**Architectural features.** Examples of the architectural feature extraction performed in this study. Shown are (**a**) the Voronoi Diagram, (**b**) Delaunay Triangulation, (**c**) Minimum Spanning Tree, and (**d**) nuclear density calculation.

We denote a graph as $\left(G=(V,E,W)\right)$, where *V * are vertices, *E* are edges, and *W * are weights of the edges, proportional to length. The set of vertices, edges, and weights make up a unique graph on $\left(R\right)$. Examples of the graphs are shown in Figure 5, while Figure 6 illustrates the graphs as they appear on tissue images. Details of graph construction are given below.

#### Voronoi diagram ($\left(G_{\text{Vor}}\right)$)

The Voronoi Diagram partitions $\left(R\right)$ into a set of non-overlapping polygons, denoted *P*_1_,*P*_2_,⋯ ,*P*_*m*_. Vertices in *V * represent the centroids of the polygons; thus *v*_1_∈*V* is the centroid for polygon *P*_1_. Non-centroid pixel *r*∈*R*is included in polygon *P*_*a*_if the following condition is satisfied:

(1) $$||r-v_{a}||=\underset{j}{\min}\{||r-v_{j}||\},$$

where *a*,*j* ∈ {1,2,⋯ ,*m*} and ||·|| is the Euclidean distance between two points. That is, pixels are assigned to the polygon of the nearest centroid. This yields a tessellation of the image, as shown in Figure 5(a). Pixels that are equidistant from exactly two centroids make up *E* (edges of the graph, shown in red), while pixels equidistant from three or more centroids make up the intersections of multiple edges. Note that in this case *V * are not the endpoints of the edges in the graph, but are the centroids around which the polygons are constructed. The perimeter, area, and chord lengths of each polygon in $\left(G_{\text{Vor}}\right)$ are computed, and the average, standard deviation, disorder^a^, and minimum to maximum ratio of each are calculated for a total of 12 Voronoi-based features per $\left(R\right)$.

#### Delaunay triangulation ($\left(G_{\text{Del}}\right)$)

The Delaunay Triangulation is a triangulation of vertices *V * such that the circumcircle of each triangle contains no other vertices. This corresponds to the dual graph of the Voronoi Diagram, meaning that centroid points *v*_*a*_ and *v*_*b*_ are connected in $\left(G_{\text{Del}}\right)$ if and only if polygons *P*_*a*_ and *P*_*b*_ share an edge in $\left(G_{\text{Vor}}\right)$. An example of $\left(G_{\text{Del}}\right)$ is given in Figure 5(b); shown faded is $\left(G_{\text{Vor}}\right)$ to illustrate the relationship between the two. In this graph, the vertices *V * constitute the endpoints of the edges *E*. From this graph, we compute the area and perimeter of each triangle, and the average, standard deviation, disorder, and minimum to maximum ratio of these are calculated to yield 8 Delaunay-based features per $\left(R\right)$.

**Figure 7:**
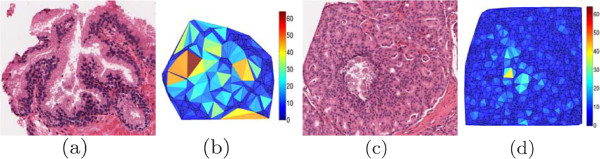
**Illustrated differences in feature values.** Illustration of the difference in feature values between two different tissue class images. Shown are (**a**) a benign epithelium tissue image with (**b**) its Delaunay triangulation, where the color of each triangle corresponds to an area value (blue represents low area, while red represents high). There is a clear difference when compared with (**c**) a Gleason grade 5 tissue sample and (**d**) its associated graph, indicating that architectural features are effective at discriminating tissue classes.

An example illustrating the Delaunay graphs of two different tissue types is shown in Figure 7, where 7(a) illustrates a benign epithelium tissue image with 7(b) its Delaunay triangulation, where the color of each triangle corresponds to an area value (blue represents low area, while red represents high). When compared with 7(c) a Gleason grade 5 tissue sample, and 7(d) its Delaunay graph, there is a clear difference in overall triangle size throughout the images.

#### Minimum spanning tree ($\left(G_{\text{MST}}\right)$)

A spanning tree is an undirected, fully connected graph on *V *. The weight *W * of the graph is the sum total of all edges *E*, and the Minimum Spanning Tree is the spanning tree with the lowest overall *W *. The Minimum Spanning Tree (MST), denoted $\left(G_{\text{MST}}\right)$, is a subgraph of the $\left(G_{\text{Del}}\right)$. An example of $\left(G_{\text{MST}}\right)$ is given in Figure 5(c); again, we superimpose $\left(G_{\text{Del}}\right)$ to show the relationship between the two. We calculate the average, standard deviation, disorder, and minimum to maximum ratio of the weights *W * to yield 4 MST-based features per $\left(R\right)$.

#### Nuclear density

Finally, we calculate a set of features that quantify the density of the nuclei without reliance on graph structures. Nuclear density features are calculated in two different ways: (1) We construct a circle around each point in *V * with a fixed radius (black circle in Figure 5(d)), and count the number of neighboring points in *V * that fall within that circle. This is done for radii of 10, 20, 30, 40, and 50 pixels, and for each point in *V *. The average, standard deviation, and disorder is computed across all points in *V * to yield 15 features for each $\left(R\right)$. (2) We calculate the distance from a point in *V * to the nearest 3, 5, and 7 neighbors (red lines in Figure 5(d)). This is done for each point in *V *, and the average, standard deviation, and disorder is computed to yield 9 additional features, for a total of 24 features based on nuclear density.

#### Image texture feature extraction

**Figure 8:**
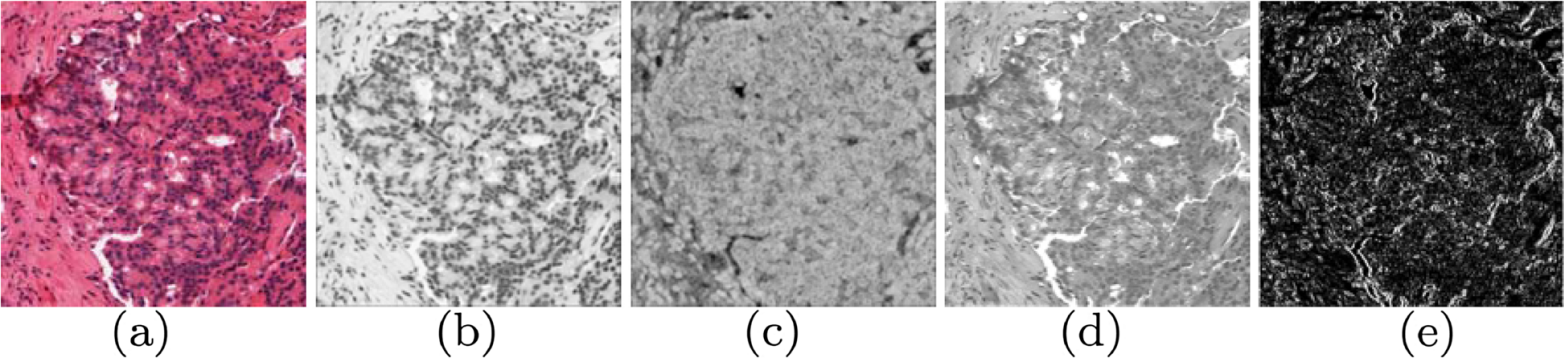
**Examples of texture features.** Examples of the texture feature images generated during feature extraction. Shown are (**a**) the original image, (**b**) first-order statistics (average intensity), (**c**) co-occurrence feature values (contrast entropy), and (**d**), (**e**) two steerable Gabor filters (*κ* = 5, $\left(\theta =\frac{5\cdot \Pi }{6}\right)$) illustrating the real and imaginary response, respectively).

The proliferation of nuclei, difference in size and shape of lumen area, and breakdown of typical glandular structure (see Figure 1) leads to a change in overall textural characteristics in an ROI. To quantify this change in tissue texture characteristics, we calculate a number of low-level image statistics from each ROI. These statistics can be broadly characterized into three groups: first-order statistics, second-order co-occurrence features, and steerable filter features. Each of these is calculated in a pixel-wise fashion and are computed independently for each of the hue, saturation, and intensity channels of the original scanned image, generating a set of feature images (Figures 8(a)-(d)). The average, standard deviation, and mode of each of these feature images is calculated, yielding a texture feature vector to quantify the image. In total, 540 texture features are calculated in this manner. The details of each feature type are given below.

#### First-order statistics

We calculate 15 different first-order statistics from each image, including average, median, standard deviation, and range of the image intensities within the sliding neighborhood, as well as the Sobel filters in the vertical, horizontal, and both diagonal axes, 3 Kirsch filter features, gradients in the vertical and horizontal axes, difference of gradients, and diagonal derivative. By calculating these 15 features for each channel in the image, and then calculating the mean, standard deviation, and mode of the feature images, we obtain a total of 135 first-order statistics for $\left(R\right)$. An example of the average hue feature image is shown in Figure 8(a).

#### Co-occurrence features

Co-occurrence features [29] are computed by constructing a symmetric 256×256 co-occurrence matrix which describes the frequency with which two different pixel intensities appear together within a fixed neighborhood. The number of rows and columns in the matrix are determined by the maximum possible value in a channel of $\left(R\right)$; for 8-bit images, this corresponds to 2^8^=256. Element (*ab*) in the matrix is equal to the number of times pixel value *a* occurs adjacent to pixel value *b* in $\left(R\right)$. From the co-occurrence matrix, a set of 21 features are calculated: autocorrelation, contrast, correlation, cluster prominence, cluster shade, dissimilarity, energy, entropy, homogeneity, maximum probability, variance, sum average, sum variance, Sum entropy, difference variance, difference entropy, two information measures of correlation, inverse difference, normalized inverse difference, and inverse moment [29,30]. Extracting these values from each channel and taking the mean, standard deviation, and mode of each feature image yields a total of 189 co-occurrence features. An example of the contrast entropy image is shown in Figure 8(b).

#### Steerable filters

A steerable filter refers to a filter which is parameterized by orientation. One such filter is the Gabor filter [31,32], which is a Gaussian function modulated by a sinusoid. The response of a Gabor filter at a given image coordinate is given as:

(2) $$G(x,y,\theta ,\kappa )=e^{-\frac{1}{2}({\left(\frac{x^{\prime }}{\sigma_{x}}\right)}^{2}+{\left(\frac{y^{\prime }}{\sigma_{y}}\right)}^{2})}\cos(2\Pi \kappa x^{\prime }),$$

where *x*^*′*^=*x*cos(*θ*) + *y*sin(*θ*), *y*^*′*^=*y*cos(*θ*) + *x*sin(*θ*), *κ* is the filter’s frequency shift, *θ* is the filter phase, *σ*_*x*_and *σ*_*y*_ are the standard deviations along the horizontal and vertical axes. We utilize a filter bank consisting of two different frequency-shift values *κ* ∈ {5,9} and six orientation parameter values ($\left(\theta =\frac{\in \cdot \Pi }{6}\right)$ where *∈* ∈ {0,1,⋯ ,5}), generating 12 different filters. Each filter yields a real and imaginary response, which is calculated for each of the three channels. An example of two Gabor-filtered images is shown in Figures 8(c) and (d), illustrating the real and imaginary response, respectively, for a filter with *κ*=5 and $\left(\theta =\frac{5\cdot \Pi }{6}\right)$. Taking the mean, standard deviation, and mode of each feature image yields a total of 216 steerable filter texture features.

### Experimental setup

#### Prostate biopsy tissue preparation, Digitization, and ROI Identification

Prostate biopsy samples were acquired from 214 patients at the Department of Surgical Pathology at the University of Pennsylvania in the course of normal clinical treatment. Tissue samples were stained with hematoxylin and eosin (H&E) to highlight nuclear and cytoplasmic material in the cells. Following fixation, the slides were scanned into a computer workstation at 40x optical magnification using an Aperio whole-slide digital scanner (Aperio, Vista, CA). The acquisition was performed following an automated focusing procedure as per the recommended software settings, and the resulting files were saved as ScanScope Virtual Slide (SVS) file format, which are similar to multi-image tagged image file format (TIFF) files. Each patient study resulted in a single image (214 images total), which contained between 2-3 tissue samples each. In terms of pixel size, each image measures from 10,000 to 100,000 pixels in a dimension, depending on the amount of tissue on the slide. Uncompressed images range from 1 gigabyte (GB) to over 20 GB in hard drive space. At the time of scanning, images were compressed using the JPEG standard to a quality of 70 (compression ratio of approximately 1:15); at the image magnification that was captured, this compression did not result in a significant loss of quality of the acquired images.

ROIs corresponding to each class of interest are manually delineated by an expert pathologist, with the goal of obtaining relatively homogeneous tissue patches (i.e. patches that express only a single tissue type). Due to the widely varied presentation of the target classes on patient biopsy, the number of ROIs obtained per patient was greatly varied (between a minimum of 5 and a maximum of 30). It should be noted that the annotation of individual tissue types on pathology is not a common practice within clinical diagnosis and prognosis of prostate biopsy samples. Thus, there are no generally-accepted guidelines for drawing exact boundaries for regions of cancer, PIN, or atrophy; however, the annotating pathologists were only told to try and ensure that the majority of each ROI was from the same tissue class. Following annotation, the images are down-sampled to a resolution equivalent to 20x optical magnification. A total of 2,256 ROIs were obtained.

#### Experiment 1: classifier comparison

Our main hypothesis is that for multi-category classification, the CAS methodology will provide increased performance when compared with the OSC and OVA strategies. The differences between each of the three strategies are summarized below: **Cascade (CAS):** The cascaded strategy is our proposed method, described in the Methods section above. **One-Shot Classification (OSC):** For the OSC strategy, the entire dataset is classified into seven classes simultaneously. This is handled implicitly by the decision tree construction, where rule branches terminate at several different class labels. **One-Versus-All (OVA):** For the OVA strategy, a binary classifier is used to identify a single target class apart from a single non-target class made up of the remaining classes. Each class is classified independently of the others, meaning that errors in one class do not affect the performance of the others.

For the binary classifier, we employed the C5.0 decision tree (DT) algorithm [22], which is an efficient update to the popular C4.5 algorithm. The motivation for using the C5.0 DT algorithm as opposed to other classifiers such as support vector machines [33], Bayesian estimators, or *k*-nearest neighbor algorithms, is its inherent ability to deal with multiple classes (by creating labeled nodes for each class), allowing us to directly compare the performance of each of the approaches described above. While other tree-based algorithms such as probabilistic boosting trees [34] possess this property as well, C5.0 is significantly faster and easier to train. We performed three-fold cross-validation for twenty trials, using approximately two-thirds of the dataset for training and one-third for testing. The output of each of the strategies consists of the number of samples from each class, and the resulting classification of those samples. This enables us to calculate the accuracy (ACC), positive predictive value (PPV), and negative predictive value (NPV) in terms of true positives (TP), true negatives (TN), false positives (FP), and false negatives (FN), where:

(3) $$\begin{array}{lll} \text{ACC} & =\frac{\mathrm{TP}+\mathrm{TN}}{\mathrm{TP}+\mathrm{TN}+\mathrm{FP}+\mathrm{FN}},\text{PPV}=\frac{\mathrm{TP}}{\mathrm{TP}+\mathrm{FP}}, & \\ \text{NPV} & =\frac{\mathrm{TN}}{\mathrm{TN}+\mathrm{FN}}. \end{array}$$

Evaluation is done on a per-class basis, to ensure that comparisons between different classification strategies were standardized.

#### Experiment 2: feature ranking

Because of the range of classes being analyzed in this work, we are interested in the discriminating power of the individual features for each classification task. This experiment is intended to provide insight into which features are contributing to the performance of each classification task. We employed the AdaBoost algorithm [35] to implicitly weight features according to their discriminating power. AdaBoost is an iterative algorithm that determines the ability of each feature to discriminate between target classes. The algorithm takes as input a parameter, *T*, which indicates how many iterations are run (and thus, how many weak learners are selected and weighted), and performs the following steps: 

1. At iteration *t*, each feature is evaluated in terms of its discriminative power for the current classification task.

2. The feature that provides the highest accuracy is selected as the *t*th iteration returned by the algorithm.

3. A weight *α*_*t*_is assigned to the selected feature, which is proportional to the feature’s discriminative power.

4. *α*_*t*_is used to modulate the performance of feature *t* in subsequent iterations, forcing the algorithm to select features which focus on correctly classifying difficult samples.

5. When *t* = *T*, the algorithm returns the set of selected features and their corresponding weights.

As the algorithm progresses, learners are selected which correctly classify samples which were misclassified by previously-selected learners. Based on the weights, we obtain a ranking of the ten most discriminating weak learners for each task, with *α*_*t*_>*α*_*t* + 1_. The obtained weights are summed across the twenty trials to obtain a final weight and ranking for the learner.

#### Experiment 3: evaluation of automated nuclei detection algorithm

Our final experiment is performed to determine whether our automated nuclear detection algorithm is accurately identifying nuclear centroids. To do this, we consider that we are not interested in perfect segmentation of nuclei, but rather a segmentation that is accurate enough to generate useful and descriptive feature values. Since exact delineation of each nuclear centroid in the image is not our main goal, traditional methods of segmentation evaluation (such as percentage overlap, Hausdorff distance, and Dice coefficients) are not appropriate for evaluating this task. To ensure that our feature extraction is performing appropriately, a subset of images from four classes (epithelium, stroma, and Gleason grades 3 and 4) had nuclear centroids manually annotated. We compared the features obtained through our automated detection algorithm, using color deconvolution and watershed segmentation, with the features obtained using manual annotation. Comparison was performed using a Student’s t-test to determine how many features had no statistically significant difference between the two sets of feature values.

The research was conducted with approval from the Institutional Review Boards at both the University of Pennsylvania and Rutgers University.

## Results and discussion

### 

#### Experiment 1: classifier comparison

**Figure 9:**
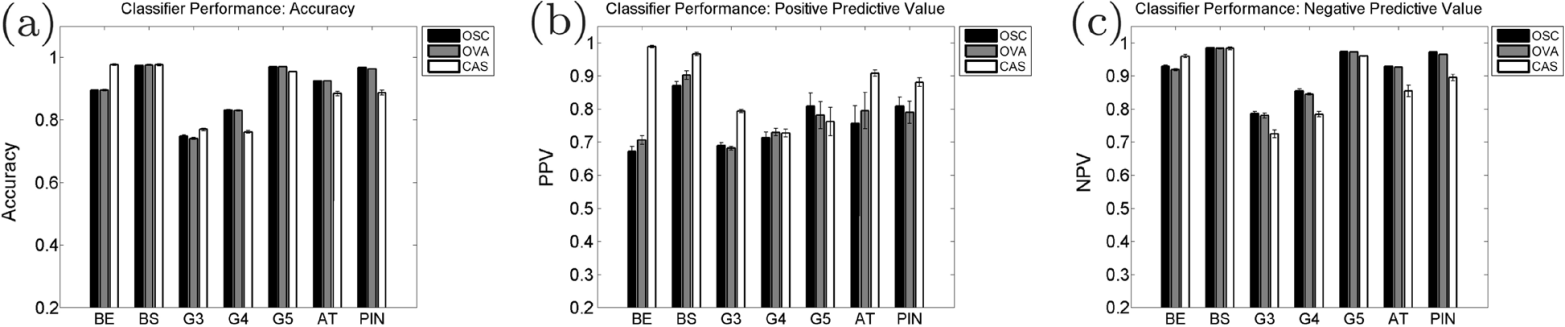
**Classifier performance for OSC, OVA, and CAS.** Average performance measures from the three different classification strategies: OSC (one-shot classification), OVA (one-versus-all classification), and CAS (our cascaded approach). Shown are the values for (**a**) accuracy, (**b**) positive predictive value (PPV), and (**c**) negative predictive value (NPV) with each group representing a separate tissue class. Error bars represent standard error over 20 trials. In terms of accuracy, the different algorithms perform similarly, with CAS showing a small advantage for most tissue classes; however, in terms of PPV, the cascaded approach out-performs both OSC and OVA in the majority of tasks, particularly with Gleason grade 3 and grade 4 tissue.

Figure 9 illustrates the performance values for each of the classification strategies. Shown are the ACC and PPV values for each of the three strategies, averaged over 20 trials (error bars represent the standard error). The average ACC across all classes is 0.90 for OSC, 0.90 for OVA, and 0.89 for CAS, while the average PPV is 0.76 for OSC, 0.77 for OVA, and 0.86 for CAS. The average NPV across all classes is 0.92 for OSC, 0.91 for OVA, and 0.88 for CAS. The quantiative results for each individual class across all strategies are given in Table 2.

**Table 2:** Quantiative classification results

		**BE**	**BS**	**G3**	**G4**	**G5**	**AT**	**PN**
	OVA	0.90	0.98	0.74	0.83	0.97	0.92	0.96
Accuracy	OSC	0.89	0.97	0.75	0.83	0.97	0.92	0.97
	CAS	0.98	0.98	0.77	0.76	0.95	0.88	0.89
	OVA	0.71	0.90	0.68	0.73	0.78	0.79	0.79
PPV	OSC	0.67	0.87	0.69	0.71	0.81	0.76	0.81
	CAS	0.99	0.97	0.79	0.73	0.76	0.91	0.88
	OVA	0.92	0.98	0.78	0.84	0.97	0.93	0.96
NPV	OSC	0.93	0.98	0.79	0.85	0.97	0.93	0.97
	CAS	0.96	0.98	0.72	0.78	0.96	0.85	0.89

Summary of quantitative results for classification according to individual classes and classifier strategies.

The CAS strategy does not out-perform the OSC or OVA strategies with respect to ACC or NPV, but there is a modest improvement in terms of PPV. The majority of errors when using the CAS approach are false positives; that is, images are more likely to identify a non-target class as the target class. However, this leads to a tradeoff in NPV, which is lower for CAS than for the alternate strategies by a small amount.

In terms of PPV, there are only two classes in which CAS is not the top-performing classification strategy: G4, which yields the same PPV as the OVA strategy, and G5, where it under-performs both strategies. These represent two very similar classes of CaP on the grading scale and are difficult to distinguish automatically [8,10]. Despite not yielding the highest PPV, the difference in the G5 class between CAS and OSC (the top-performing strategy) is 0.05.

#### Experiment 2: feature ranking

**Figure 10:**
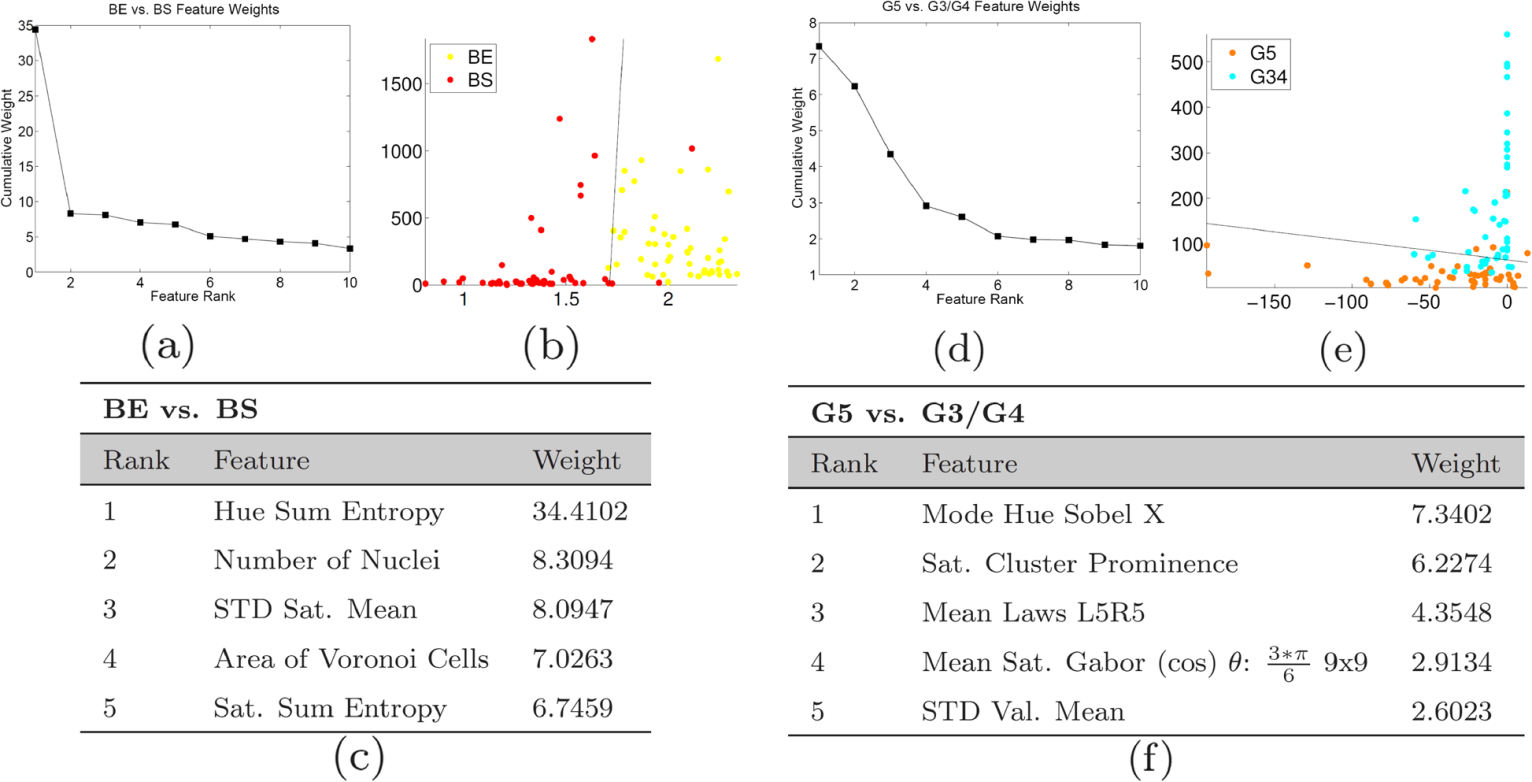
**Results of feature selection - BE vs. BS and G5 vs. G3/G4.** Results of feature selection when distinguishing between Benign Epithelium (BE) vs. Benign Stroma (BS) on the left, and Gleason grade 5 (G5) vs. Gleason grades 3 and 4 (G3/G4) on the right. Shown are: (**a**), (**d**) the plots of the cumulative weights as a function of feature rank, (**b**), (**e**) scatter plots of the first and second features along with the optimal discriminating hyperplane, and (**c**), (**f**) a list of the feature names associated with each rank.

**Figure 11:**
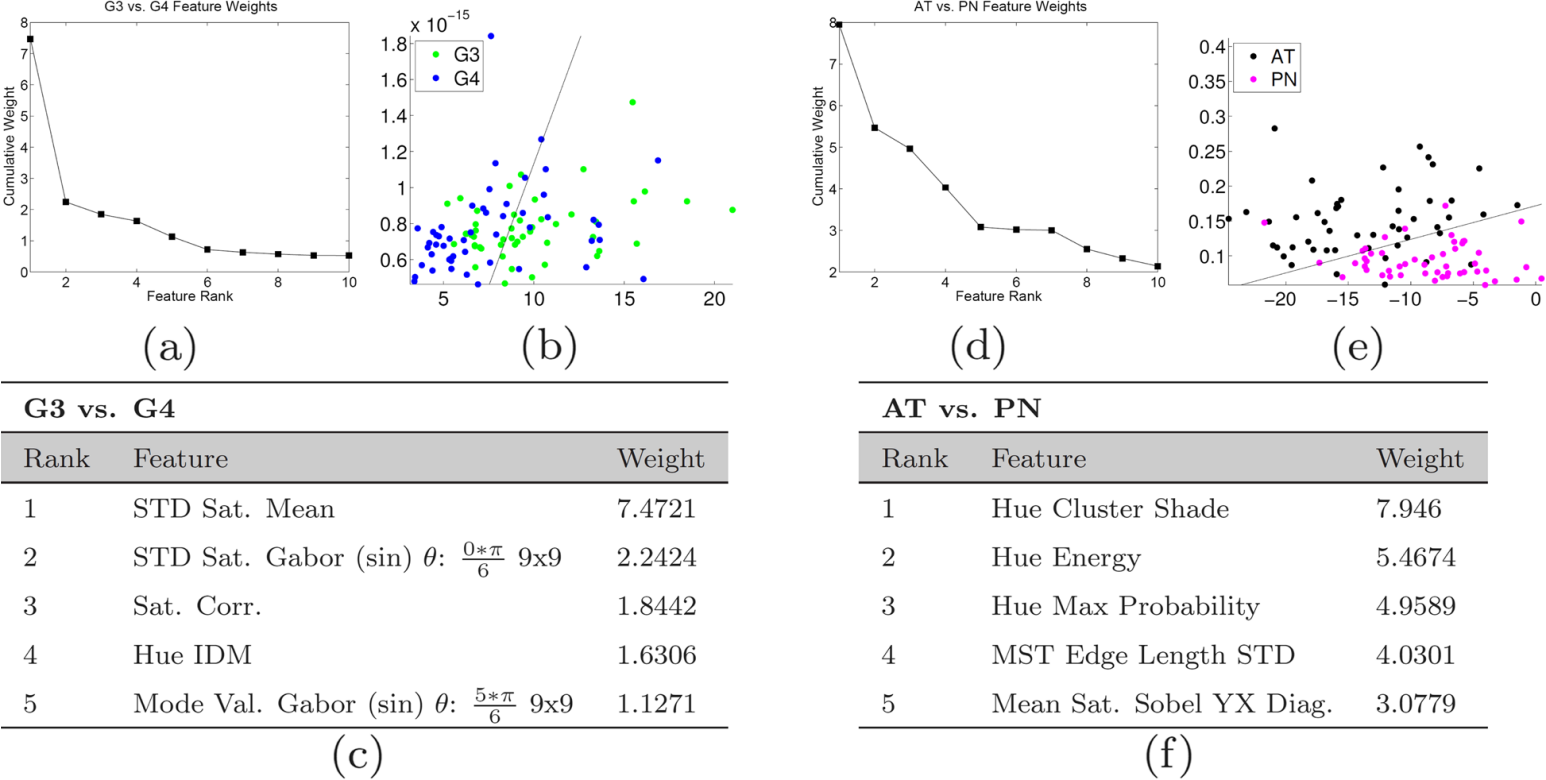
**Results of feature selection - G3 vs. G4 and AT vs. PIN.** Results of feature selection when distinguishing between Gleason grade 3 (G3) vs. Gleason grade 4 (G4) on the left, and Atrophy (AT) vs. PIN on the right. Shown are: (**a**), (**d**) the plots of the cumulative weights as a function of feature rank, (**b**), (**e**) scatter plots of the first and second features along with the optimal discriminating hyperplane, and (**c**), (**f**) a list of the feature names associated with each rank.

The results of feature ranking via AdaBoost are shown in Figure 10 for BE vs. BS and G5 vs. G3/G4 tasks, and in Figure 11 for the G3 vs. G4 and AT vs. PN tasks. Figures 10(a), (d) and 11(a), (d) contain the cumulative weights plotted as a function of rank. As more weak learners are selected, each subsequent learner has a lower weight and hence the discriminative power of each feature and its influence on the classifier decreases. Figures 10(b), (e) and 11(b), (e) illustrate a scatter plot of the data points where the horizontal and vertical axes are the feature values of the first- and second-ranked features, respectively. For each classification task, points representing the two classes are plotted with a decision boundary that optimally separates the classes. These plots illustrate the separation between each set of classes using only the features used in the first two selected weak learners. Figures 10(c), (f) and 11(c) and (f) show the names of the features used in the top five ranked weak learners. The weights for all classification tasks drop rapidly and level off after approximately 10 features are chosen, indicating that a small subset of the entire feature set is able to perform adequately in discriminating between each class.

In distinguishing different grades of cancer (G5 vs. G3/G4 and G3 vs. G4), all of the top five selected features are texture-based features. The subtle differences between Gleason grades of prostate tissue are not picked up by quantitative architecture, as the biological variation in the features likely eliminates any discriminating power these features have. The more granular texture features, however, are capable of identifying these subtle changes in nuclear proliferation and lumen area which are major indicators of progressing disease.

For the non-cancer tasks – BE vs. BS, and AT vs. PN – we find that both architectural and textural features are in the top-ranked features. This can be appreciated by referring to the examples of tissue shown in Figure 1 as well as the architectural heat map in Figure 7. In both sets of non-cancer classification tasks, the target classes have either large, well-organized glandular structures (BE and AT) or sparse, less-structured tissue types with fewer arranged nuclei (BS and PN). Architectural features are well-suited to quantify the differences represented by these large structures, and so we see these features receiving higher weight than they do when distinguishing Gleason grades.

#### Experiment 3: evaluation of automated nuclei detection algorithm

The results of comparing the feature sets generated via manual and automated nuclei detection are shown in Table 3. For each of the four classes with manually-annotated nuclei, we list how many features had *p* > 0.05 and *p* > 0.01, indicating that there was no statistically significant difference between the manually- and automatically-extracted features. We found that at least 9 features (out of the 51 total architectural features) were considered statistically similar in all classes, with Gleason grade 5 and stroma having the most (over 20) similar features. This is likely due to the lack of complex structure (such as lumen and intra-luminal protein), enabling the automated system to clearly single out the nuclei in the image. In contrast, Gleason grade 3 had the fewest similar features due to the high degree of proliferation of cancer and the presence of gland structures, which leads to a high number of adjacent and overlapping nuclei. These centroids are difficult to correctly identify both manually and algorithmically, so the greatest amount of disagreement is seen in this class. In general, Voronoi features tended to be significantly similar between the two methods, while nuclear density features (which are highly sensitive to false-positive nuclear segmentations) had the least similarity.

**Table 3:** Statistical differences between automatic and manual architectural features

**Class**	*p***> 0.05**	*p***> 0.01**
Epithelium	13	17
Stroma	24	26
Grade 3	6	9
Grade 4	11	15
Grade 5	28	29

Number of architectural features whose values were considered statistically similar between automatically- and manually-detected nuclei by two different criteria (*p*>0.05 and *p*>0.01). Stroma and Gleason grade 5 tissue yielded the most similar features, while Gleason grade 3 had the lowest number of similar features.

**Figure 12:**
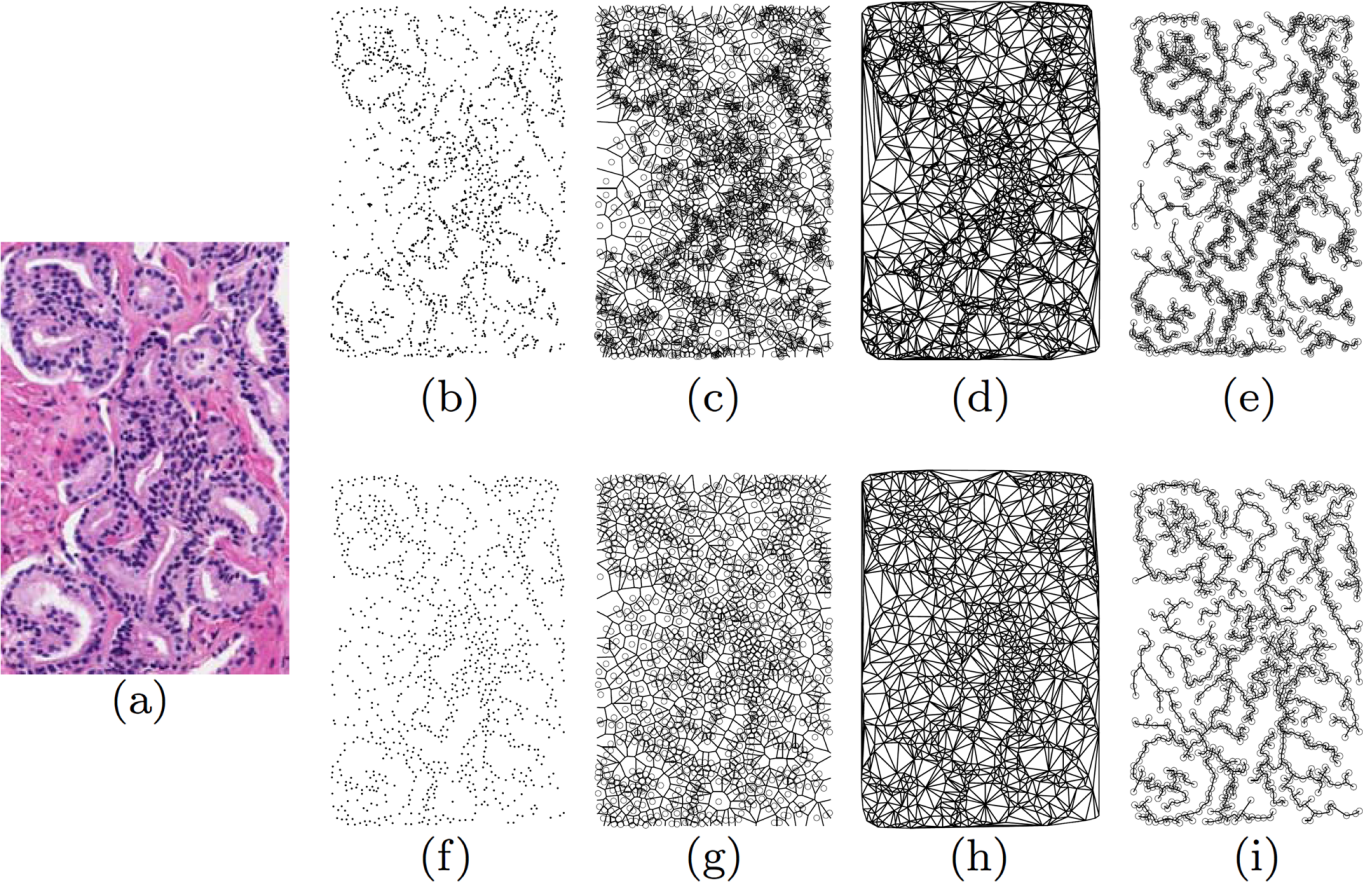
**Graphs representing automated vs. manual nuclei detection.** Examples of feature images obtained for a Gleason grade 3 image via manual (**b**)-(**e**) and automated (**f**)-(**i**) nuclear annotations. Shown are the original image at left, followed by the nuclear locations ((**b**), (**f**)), Voronoi diagrams ((**c**), (**g**)), Delaunay triangulation ((**d**), (**h**)), and minimum spanning trees ((**e**), (**i**)). Although the automated annotation tends to pick up multiple false positives, the feature values listed in Table 3 indicate that the differences are not statistically significant for each image class.

Shown in Figure 12 are representative graph images of G3 tissue obtained via automated nuclei detection (top row, Figures 12(b)-(e)) and manual annotation (bottom row, Figures 12(f)-(i)). The qualitative similarity between the manual and automatically-extracted graphs indicates that the automated method will result in feature values similar to the manual method.

## Conclusions

In this work, we have presented a cascaded multi-class system that incorporates domain knowledge to accurately classify cancer, non-cancer, and confounder tissue classes on H&E stained prostate biopsy samples. By dividing the classification into multiple class groups and performing increasingly granular classifications, we can utilize *a priori* domain knowledge to help tackle difficult classification problems. This cascaded approach can be generalized to any multi-class problem that involves classes which can be grouped in a way that maximizes intra-group homogeneity while maximizing inter-group heterogeneity. We have developed a set of quantitative features that can accurately characterize the architecture and texture of prostate biopsy tissues, and use this information to discriminate between different tissue classes. We have shown that our automated nuclei detection algorithm generates feature values which are comparable to those obtained by manual delineation of nuclei, a more appropriate evaluation of detection than a point-by-point comparison between the two methods. Finally, we analyzed the discriminating power of each of our features with respect to each classification task in the cascade, and we found that for class groups with highly structured tissues, architecture plays an important role; however, in cases where tissue types are very similar (i.e. distinguishing Gleason grade), texture is more important to capture the subtle differences in tissue structure.

In our current implementation of the CAS approach, we made the assumption that domain knowledge should be the driving force behind the order of the cascaded classifiers. However, this may not be optimal, and other cascaded setups could also be used. For example, we would calculate an image metric from the training data that would allow us to divide the data into homogeneous groups based on the feature values, thus further separating the classes in each task. Using a proper distance metric to drive the initial design of the system might increase the classifier’s overall performance. In addition, we would like to investigate the use of alternative classification algorithms capable of performing one-shot classification, such as neural networks.

## Endnotes

^a^For a feature with standard deviation *A* and mean *B*, the disorder is calculated as: $\left(1-\frac{1}{1+\frac{A}{B}}\right)$.

## Competing interests

Scott Doyle and Anant Madabhushi are both equity holders in Ibris, Inc.

## Author’s contributions

SD developed and tested the algorithms, processed the results and wrote the manuscript. JT and MF provided the dataset, annotations, and medical information and guidance. NS performed tissue scanning, quality control, and annotation of the image data. AM directed the research and the development of the manuscript. All authors read and approved the final manuscript.
